# Evaluation of the efficacy of a sunscreen containing ultra‐long UVA1 and other UVR broad‐spectrum filters on skin barrier protection and melanin content reduction in Chinese adults: A single‐center study

**DOI:** 10.1002/hsr2.1923

**Published:** 2024-02-22

**Authors:** Xianghua Zhang, Han Tao, Zhongxing Zhang, Wenna Wang, Andrew Steel, Xiaofeng Fang, Xiaofeng He

**Affiliations:** ^1^ Dermatological Beauty Division, L'Oréal China Shanghai China; ^2^ China Research and Innovation Center, L'Oréal China Shanghai China

**Keywords:** Mexoryl® 400, skin barrier, skin pigmentation, UVA1, Vitamin E

## Abstract

**Background and Aims:**

The protection for ultra‐long UVA1 is lacked in the market, posing potential damage from ultra‐long UVA1 irradiation. The study aims to evaluate the efficacy of a sunscreen containing multiple components, especially Mexoryl® 400 for improving skin barrier function and reducing melanin content.

**Methods:**

This single‐center study included adults with sensitive and normal skin in China in November 2022. Participants received the test sunscreen for 4 weeks. Melanin and hemoglobin content, sebum secretion skin hydration, and trans‐epidermal water loss were evaluated at T0d, T7d, and T28d. The self‐assessment was done at T15min, T7d, and T28d.

**Results:**

Sixty participants were included, including 30 self‐claimed sensitive skin in the sunscreen group. The sunscreen demonstrated significant improvements in skin parameters. Skin redness reduced by 9.84% at T28d, sebum content in the forehead area decreased by 22.70% at T28d, and skin stratum corneum hydration increased by 38.44% at T28d, *p* < 0.001 respectively. Most notably, skin melanin content significantly reduced by 13.49% after 4 weeks' usage (*p* < 0.001). No adverse reactions were reported in either group.

**Conclusions:**

The study sunscreen improved the skin condition by decreasing the melanin content, regulating skin barrier function, and achieving a balance of skin hydration and sebum secretion.

## 1 INTRODUCTION

Solar radiation, specifically ultraviolet (UV) radiation, can cause significant damage to the skin. Among the UV radiation (UVR) that can penetrate the atmosphere, UVB accounts for 5% while UVA accounts for 95%.^1^ Our skin is exposed to more UVA (320−400 nm) than UVB (290−320 nm). UVA is further divided into UVA2 (shortwave: 320−340 nm) and UVA1 (longwave: 340−400 nm).^2^ Both UVA and UVB can potentially induce DNA photoproducts that lead to erythema, pigmentation, and other skin issues. Common exposure to UVR can also influence the skin barrier function.^3^

Pigmentation is a major concern caused by UVR, which can cause psychological and emotional distress.^4^ The pigmentation can be caused both by UVA and UVB. Exposure to UVB can induce delayed tanning while exposure to UVA can induce immediate pigment darkening followed by persistent pigment darkening.^5^ The pigmentation induced by UVB exposure occurs in the superficial layer of the skin while exposure to UVA leads to an increase in melanin content in the deeper layers of the epidermis.^6^ Specifically, UVA‐induced melanin accumulation is wavelength dependent: UVA1 increases melanin density in the basal cell layer, while UVA2 increase the number of melanin granules in the more superficial layer of the epidermis.^7^

Given the current state of sunscreen technology, a combination of sun filters must be chosen to provide photostable, high‐SPF, and broad‐spectrum protection to consumers.^8^ Therefore, sunscreens that cover a wider range of the UVB and UVA spectra are considered to have higher sun protection capabilities. However, the protection against ultra‐long UVA1 (370−400 nm) in the market is rarely addressed due to a lack of relevant UV absorbers.

It was reported that Mexoryl® 400 (Methoxypropylamino Cyclohexenylidene Ethoxyethylcyanoacetate) can cover UVA1 in the range of 380−400 nm. The compound has been shown to reduce in vitro biological alterations and in vivo induced pigmentation under UVA1 exposure.^9^ In combination with other sun filters and vitamin E, the sunscreen would be photostable, with broad‐spectrum protection and high SPF.^8^ Vitamin E also has antioxidant and skin barrier stabilizing properties.^10^

Based on the above, this single‐center study aimed to evaluate the effectiveness of a sunscreen containing ultra‐long UVA1 filter and other sun filters combined with vitamin E in treating pigmentation disorders and barrier function of the skin by providing full‐spectrum protection against UVR.

## 2 METHODS

### 2.1 Study design and subjects

This single‐center study conducted by Societe Generale de Surveillance S.A., (reference number: SHCPCH221108560) in Hangzhou China, enrolled 60 adult participants aged 18−44 years (19 males and 41 females) with various skin types. To qualify as having sensitive skin, participants completed a comprehensive questionnaire which included questions about historical skin conditions like eczema or dermatitis, as well as recent skin reactions to environmental factors, cosmetics, dietary components, and stress. This self‐assessment helped classify at least 30 individuals with sensitive skin based on criteria such as intolerance to temperature changes, reactivity to pollution, and adverse reactions to cosmetics or emotional stress. Enrollment began 2 weeks before the study, during which time subjects were required to cease all use of sunscreen. Baseline skin measurements were conducted before the sunscreen cessation.

Inclusion criteria were: (1) Chinese male or female aged 18−45 years; (2) good overall health with no physical, mental, or social diseases; (3) willingness to sign an informed consent and ability to read, speak, write, and understand Mandarin; and (4) no facial treatment in the past 6 months and willingness to stop all facial treatments during the study.

Key exclusion criteria were: (1) use of makeup (foundation, BB cream, etc.) and sun protection products within 2 weeks before the study; (2) history of allergy to facial care products; (3) visible health conditions and/or skin diseases that may interfere with study results; (4) pregnancy or planning to become pregnant; (5) history of skin cancer within the past 5 years; (6) cardiovascular, cerebral, pulmonary, hepatic, or renal diseases; and (7) psychological or psychiatric diseases. Subjects were evaluated by a qualified medical professional to ensure they did not have any health conditions or diseases that would interfere with the study.

### 2.2 Intervention

Participants followed a designated skincare routine for the duration of the study. Each morning, they cleansed their face using the provided standard cleanser (La Roche‐Posay purifying foaming cream) and applied their regular, non‐active ingredient moisturizer. Following this, they applied the test sunscreen to their entire face. The sunscreen was reapplied in the afternoon, using a coin‐sized amount each time. In the evening, participants cleansed their face again with the standard cleanser and applied their regular moisturizer.

### 2.3 Instrumental assessment

Melanin and hemoglobin content in the skin, primarily responsible for skin color, were measured using a Mexameter MX 18 (Courage + Khazaka electronic GmbH), which emits light at three predefined wavelengths and measures the light reflected by the skin with a specialized probe. The positioning of the emitter and receiver ensures that only diffuse and scattered light is measured, allowing for an accurate assessment of skin pigmentation and erythema levels.

Sebum secretion of the forehead skin was measured using a Sebumeter® SM815 (Courage + Khazaka electronic GmbH), which is based on grease spot photometry.

Skin hydration was assessed using a Corneometer CM825 (Courage & Khazaka), which employs capacitance to test the skin's surface moisture content. The capacitive measurements were conducted in arbitrary units (a.u.) by applying a probe consisting of a condenser (7 × 7 mm) to the skin surface under constant pressure (3.5 N).

Transepidermal water loss (TEWL) was evaluated using a Tewameter TM Hex (Courage & Khazaka), which measures the passive transfer of water through the stratum corneum. The TEWL measurement is based on the diffusion principle in an open chamber, with a density gradient indirectly measured by two pairs of sensors located within a hollow cylindrical probe.

Facial images were captured using a VISIA imaging system (Canfield Imaging Systems) equipped with a Canon Mark II digital camera (Canon Incorporated). Images were taken of each participant's face (left side, front, right side) under various light sources, such as standard light, UV light, and cross‐polarized light sources.

### 2.4 Self‐assessment

A self‐assessment questionnaire of product efficacy evaluation was filled out by each subject at T15min, T7d, and T28d after using the sample; a self‐assessment questionnaire of product sensory evaluation was filled out by each subject at 4 weeks after using the sample. Scores for each question were 1−5 points: 5, agreement; 4, relative agreement; 3, neutrality; 2, relative disagreement; 1, disagreement. The proportion of subjects with 4 or 5 points for each question was calculated.

### 2.5 Statistical analysis

Statistical analysis was performed with IBM SPSS Statistics 29. Continuous data with normal distribution were expressed as the number of valid participants, mean, standard deviation, minimum, maximum, and median values at each time point, and compared between the two groups by the Student's *t*‐test. Continuous data with non‐normal distribution were expressed as median (interquartile range) and analyzed by nonparametric tests. Repeated measured continuous data were analyzed by the Wilcoxon signed‐rank test or paired *t*‐test, depending on the normality analysis results. A two‐sided *p* Values less than 0.05 was considered to indicate statistical significance. For precision, *p* Values are reported to three decimal places; values less than 0.001 are reported as <0.001.

## 3 RESULTS

### 3.1 Baseline patient characteristics

A total of 67 subjects were screened and met the eligibility criteria. Then, 67 participants were enrolled. Seven subjects voluntarily withdrew from the study without follow‐up. Finally, 60 subjects were included in the analysis.

The maximum age was 44 and the minimum age was 18. The mean age of the participants was 31.00 ± 7.32‐year‐old, and 30 individuals self‐assessed as having sensitive skin.

### 3.2 Treatment efficacy

After 1 week of treatment, the skin redness a* value, as captured by VISIA, was significantly reduced by 6.50% compared to baseline. After 4 weeks of treatment, the skin redness a* value was further significantly reduced by 9.84% (T0d: 25.31 ± 6.47, T7d: 23.67 ± 6.35, T28d: 22.82 ± 6.99, with *p* < 0.001 respectively). The sebum content in the forehead area, as measured by Sebumeter, was significantly reduced by 13.11% after 1 week of treatment and by 22.70% after 4 weeks (T0d: 199.11 ± 49.35, T7d: 173.00 ± 44.29, T28d: 153.92 ± 41.59, with *p* < 0.001 respectively).

The TEWL rate, as measured by Tewameter, was significantly reduced by 6.81% after 1 week of treatment and by 12.74% after 4 weeks (T0d: 21.59 ± 4.03, T7d: 20.12 ± 4.01, T28d: 18.84 ± 3.44, with *p* < 0.001 respectively). The skin stratum corneum hydration, as measured by Corneometer, was significantly increased by 16.53% after 1 week of treatment and by 38.44% after 4 weeks (T0d: 33.15 ± 10.31, T7d: 38.63 ± 10.07, 4 weeks: 45.89 ± 9.29, with *p* < 0.001 respectively).

The skin hemoglobin content, as measured by Mexameter, was significantly reduced by 5.85% after 1 week of treatment and by 9.64% after 4 weeks (T0d: 320.81 ± 59.24, T7d: 302.03 ± 55.03, T28d: 289.88 ± 55.15, with *p* < 0.001 respectively). The skin melanin content, as measured by Mexameter, was significantly reduced by 5.00% after 1 week of treatment and by 13.49% after 4 weeks (T0d: 134.65 ± 34.44, T7d: 127.92 ± 32.36, T28d: 116.49 ± 30.87), with *p* < 0.001, respectively.

**Figure 1:**
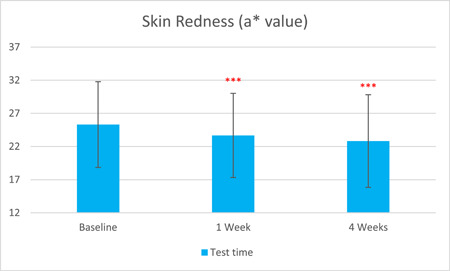
Skin Redness results from T0d to T28d.

**Figure 2:**
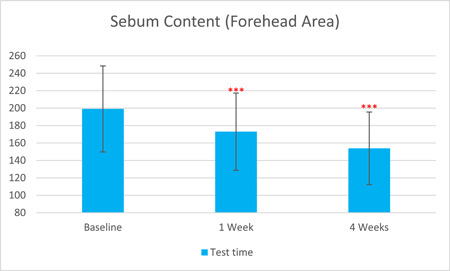
Skin sebum content results of forehead area from T0d to T28d.

**Figure 3:**
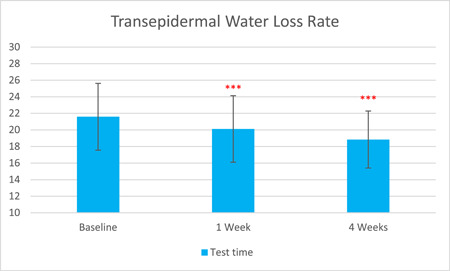
Skin transepidermal water loss rate results from T0d to T28d.

**Figure 4:**
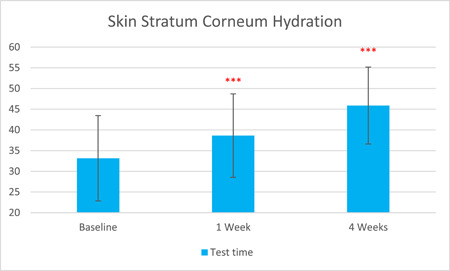
Skin stratum corneum hydration results from T0d to T28d.

**Figure 5:**
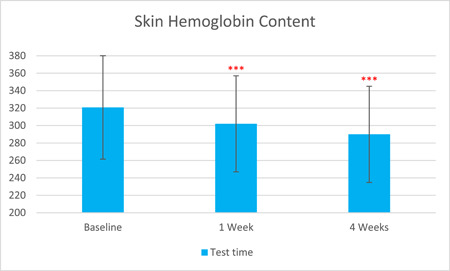
Skin hemoglobin content results from T0d to T28d.

The results are shown in Figure 1, 2, 3, 4, 5, and 6. One average case with VISIA pictures is shown in Table 1.

**Figure 6:**
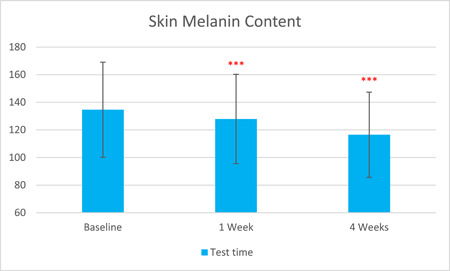
Skin melanin content results from T0d to T28d.

**Table 1:** VISIA pictures of an average case from T0d to T28d.

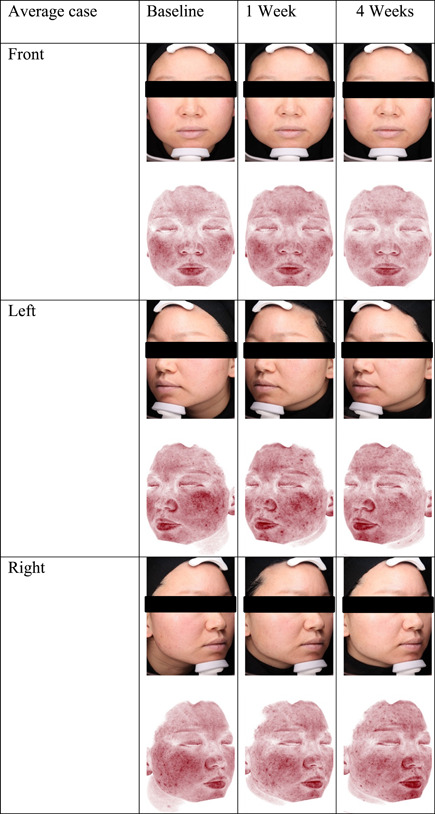

### 3.3 Self‐assessment

After 15 min of use, all participants reported the product as easy to use, smooth, and evenly spreadable, with 98% noting the quick formation of a protective film. The product was also unanimously described as lightweight, nongreasy, non‐sticky, and compatible with skin tone. Following 4 weeks of consistent use, the product sustained high satisfaction levels, with 98% of participants indicating a willingness to purchase it, regardless of price. When asked to reflect on their experiences with similar products used before the study, 37% of participants perceived the test product as far superior, 48% rated it slightly better, 13% found it comparable, and 2% felt it was slightly inferior, with none considering it significantly worse.

## 4 DISCUSSION

This single‐center study evaluated the efficacy, safety and tolerance of a sunscreen containing multiple components, including Mexoryl® 400, avobenzone, Univul® A + , Mexoryl® SX, Tinosorb® S, Mexoryl® XL, octylsalicylate, Uvinul® T150, La Roche‐ Posay thermal spring water, glycerin, vitamin E, for improving the skin condition in adults with sensitive and normal skin. Instrumental methods and self‐assessment were used to evaluate the skin response to the sunscreen.

It was found that the skin melanin content, the skin redness a* value, the skin hemoglobin content, sebum secretion, and skin hydration were improved, mainly starting after 1 week's usage. These results suggested that the study sunscreen, was very effective against UVR and could repair the skin barrier. Various studies have demonstrated the efficacy of commonly used UVA filters, including avobenzone, Univul® A+, Mexoryl® SX, Tinosorb® S and Mexoryl® XL,^8^ combined with Mexoryl® 400, supporting the current results. Besides, the study sunscreen contained octylsalicylate and Uvinul® T150, which are effective UVB filters. In addition, the sunscreen contains vitamin E. The antioxidant and photoprotective properties of vitamin E as well as its sunscreening properties^11^ have already been tested in sunscreens. Therefore, vitamin E is beneficial for both improving the skin barrier and the sunscreen efficacy.

The sunscreen's ability to reduce melanin content can be attributed to its comprehensive formulation, which includes a range of UVA filters, particularly Mexoryl® 400, an ultra‐long UVA blocker that effectively protects the skin against UVA radiation within the 370−400 nm range.^9^ UVA radiation, especially within the UVA1 range (340−400 nm), penetrates deep into the skin and can cause an increase in melanin production, leading to hyperpigmentation. While commonly used UVA filters such as avobenzone and Tinosorb® S offer protection mainly in the UVA2 range, Mexoryl® 400 fills a critical gap by extending the protection to the 380−400 nm range. Studies have shown that this ultra‐long UVA1 protection is crucial for preventing hyperpigmentation and other forms of UVA‐induced skin damage.^2^ By including Mexoryl® 400 in the formulation, the sunscreen effectively targets this ultra‐long UVA1 range, reducing melanin content by preventing the skin's exposure because UVA1‐induced pigmentation is high in a short time and can persist for weeks.^2^

The data in the study indicate that the broad‐spectrum sunscreen is effective in enhancing the skin barrier function. A notable reduction in TEWL was observed, suggesting that the sunscreen aids in preserving the skin's natural moisture levels, contributing to better hydration and, consequently, overall skin health. By preventing excessive water loss, the sunscreen effectively strengthens the skin's barrier, making it more resilient.

Moreover, the sunscreen's comprehensive protection against the full spectrum of UVA and UVB radiation further supports its role in maintaining a healthy skin barrier. Previous studies have reported the detrimental effects of UVB radiation, such as delayed alterations and epidermal proliferative responses, on the skin barrier.^12^ Additionally, prolonged exposure to UVR, particularly UVA1, can damage structural proteins like collagen and elastin, thereby compromising the skin's barrier function.^13^ The sunscreen's ability to shield the skin from such harmful UVR is crucial in preventing this damage and sustaining the integrity of the skin barrier.

The efficacy of the sunscreen in improving the skin barrier function is multifaceted. It not only helps to reduce TEWL and preserve the skin's natural moisture levels but also protects the skin from UVR‐induced damage.^14^

It should be noted that the study being conducted during winter, when UVR levels are generally lower than in summer,^15^ adds an interesting aspect to the evaluation of the sunscreen's efficacy. Despite the lower UVR levels, the broad‐spectrum sunscreen demonstrated significant improvements in various skin parameters, including reducing redness, controlling sebum production, enhancing skin barrier function, increasing hydration, alleviating inflammation, and reducing hyperpigmentation.

The fact that the sunscreen exhibited such positive results during winter highlights the importance of using sunscreen not only during summer months but also throughout the year, as UVR can still have an impact on skin health, even during periods of lower exposure. The sunscreen's effectiveness in the winter also implies that its performance in periods of higher UVR, such as summer, could be even more beneficial in preventing skin damage. It emphasizes the sunscreen's efficacy in various conditions and encourage the consistent use of sunscreen to protect and improve skin health, regardless of the season.

However, this study does have limitations that should be considered. The timing of the study during the winter months could affect the applicability of the results to periods of higher UVR exposure, such as the summer months. Also, the single‐center approach may not fully represent the broader population and diverse environmental conditions. While a 4‐week follow‐up period is sufficient to demonstrate the sunscreen's protective efficacy, it is less conclusive regarding the potential cumulative benefits of long‐term use, which could be further explored in future studies.

In conclusion, using the study sunscreen for 4 weeks improved the skin condition by decreasing the melanin content and regulating skin barrier function, achieving a balance of skin hydration and sebum secretion. The product has a broader protection with the newly introduced ultra‐long UVA1 blocker: Mexoryl® 400. According to self‐assessment by participants, the test product is comfortable for daily use.

## AUTHOR CONTRIBUTIONS

**Xianghua Zhang**: Conceptualization; investigation; methodology; data curation; supervision. **Han Tao**: Writing—original draft; writing—review and editing; data curation. **Zhongxing Zhang**: Methodology; investigation. **Wenna Wang**: Methodology; investigation. **Andrew Steel**: Investigation; methodology. **Xiaofeng Fang**: Investigation. **Xiaofeng He**: Investigation. All authors have read and approved the final version of the manuscript.

## CONFLICT OF INTEREST STATEMENT

The author(s) are affiliated with L'Oreal (China) and L'Oreal R&I. The remaining author declare no conflict of interest.

## TRANSPARENCY STATEMENT

The lead author Xianghua Zhang affirms that this manuscript is an honest, accurate, and transparent account of the study being reported; that no important aspects of the study have been omitted; and that any discrepancies from the study as planned (and, if relevant, registered) have been explained.

## Data Availability

The data that support the findings of this study are available on request from the corresponding author. The data are not publicly available due to privacy or ethical restrictions. Xianghua Zhang had full access to all of the data in this study and takes complete responsibility for the integrity of the data and the accuracy of the data analysis.
